# Shapes of distal tibiofibular syndesmosis are associated with risk of recurrent lateral ankle sprains

**DOI:** 10.1038/s41598-017-06602-4

**Published:** 2017-07-24

**Authors:** Qingjun Liu, Bin Lin, Zhimin Guo, Zhenqi Ding, Kejian Lian, Dasheng Lin

**Affiliations:** 1 Orthopaedic Center of People’s Liberation Army, the Affiliated Southeast Hospital of Xiamen University, Zhangzhou, China; 2 Experimental Surgery and Regenerative Medicine, Department of Surgery, Ludwig-Maximilians-University (LMU), Munich, Germany

## Abstract

Distal tibiofibular syndesmosis (DTS) has wide anatomic variability in depth of incisura fibularis and shape of tibial tubercles. We designed a 3-year prospective cohort study of 300 young physical training soldiers in an Army Physical Fitness School. Ankle computed tomography (CT) scans showed that 56% of the incisura fibularis were a “C” shape, 25% were a “1” shape, and 19% were a “Г” shape. Furthermore, we invited a randomly selected subcohort of 6 participants in each shape of DTS to undergo a three-dimensional (3D) laser scanning. The “1” shape group showed widest displacement range of the DTS in the y-axis, along with the range of motion (ROM) on the position more than 20° of the ankle dorsiflexion, inversion and eversion. During the 3-year study period, 23 participants experienced recurrent lateral ankle sprains. 7 cases of the incisura fibularis were “C” shape, 13 cases were “1” shape, and 3 cases were “Г” shape. The “1” shape showed highest risk among the three shapes in incident recurrent lateral ankle sprains. We propose that it is possible to classify shapes of DTS according to the shapes of incisura fibularis, and people with “1” shape may have more risk of recurrent lateral ankle sprains.

## Introduction

Ankle sprains are very common sports injuries but can also happen during daily activities such as walking or even getting out of bed^1, 2^. Patients with inversion ankle sprains constitute a large percentage of these injuries, and occur with an incidence of one sprain per 10,000 people per day^3, 4^. The most common mechanism of this injury is when the foot undergoes an inversion moment with the ankle in plantar flexion, damaging the lateral ligament complex of the ankle^5, 6^. The most pervasive predisposition to suffering a lateral ankle sprain is the history of at least one previous ankle sprain^7, 8^. One report showed that as much as 73% of all athletes had recurrence ankle sprains^9^.

The ankle complex comprises three articulations: the tibiotalar joint, the distal tibiofibular syndesmosis (DTS) and the subtalar joint. The talocrural joint receives ligamentous support from a joint capsule and several ligaments, including the anterior talofibular ligament (ATFL), calcaneofibular ligament (CFL), posterior talofibular ligament (PTFL), and deltoid ligament^7^. The ATFL prevents anterior displacement of the talus from the mortise and excessive inversion and internal rotation of talus on the tibia^10, 11^. The CFL restricts excessive inversion and internal rotation of the rearfoot^10, 11^. The PTFL provides restraint to both inversion and internal rotation of the loaded tibiotalar joint^10^. The ATFL is the first ligament to be damaged during a lateral ankle sprain^12^.

The DTS is a micro-movement joint. It will change in the movement of the ankle motion in relation to displacement and rotation of the DTS in the physiological state^13–18^. The pattern of the range of motion (ROM) of the DTS is a composite rotational and translational motion in the x, y and z axis, including up-and-down, back-and-forth, rotation and lateral movements^19–21^. During ankle plantar flexion and dorsiflexion, some movement normally occurs at the DTS. When the foot is moved from a plantar-flexed position to a dorsiflexed position, the joint permits approximately 1 to 2 mm of widening at the mortise^22–25^. While in the incisura fibularis, the fibula rotates around its vertical axis when the ankle is plantar flexed and dorsiflexed. Lateral fibular rotation is approximately 3° to 5° with dorsiflexion, and medial rotation is 3° to 5° with plantar flexion^23, 26, 27^. Normal motion of the ankle requires rotation, translation, and inferior/superior motion of the fibula at the syndesmosis to accommodate the trapezoidal shape of the talus.

As far as we know, the DTS has wide anatomic variability in the depth of the incisura fibularis, and the shape of the tibial tubercles^28, 29^. It was hypothesized that the width of the ankle mortise would vary with the different depth of the incisura fibularis and shape of the tibial tubercles during the changes of gait cycle, and the shape of the DTS could be involved in the risk of recurrent lateral ankle sprains. The objectives of this study were to investigate associations between shape of the DTS and risk of recurrent lateral ankle sprains.

## Materials and Methods

### Ethics Statement

The experiments were carried out in accordance with the guidelines of the Declaration of Helsinki. All experimental protocols were approved by our institutional review board (Xiamen University Ethical Review Committee, Ethics number: EC-12-007), and written informed consent was obtained from all study participants. A population based cohort in the Army Physical Fitness School was established in March 2012. All 300 young physical training soldiers, with normal ankles, were followed for a longitudinal and prospective cohort study.

### Computed tomography (CT) scans for measuring shapes of DTS

All 300 young physical training soldiers, with normal ankles, underwent CT scans by a single medical imaging technician for measuring the shapes of the DTS. None of the young physical training soldiers had medical history relevant with the ankle joint. The medical imaging technician selected the right/left ankle of the participants based on randomized numbers generated by sealed-envelope method. The shape of the DTS was assessed in the third section proximal to the tibial plafond. This section was 3 mm thick and 9 mm proximal to the tibial plafond^28, 30, 31^.

### Three-dimensional (3D) laser scanning for micro-movement characteristic of DTS

**Figure 1:**
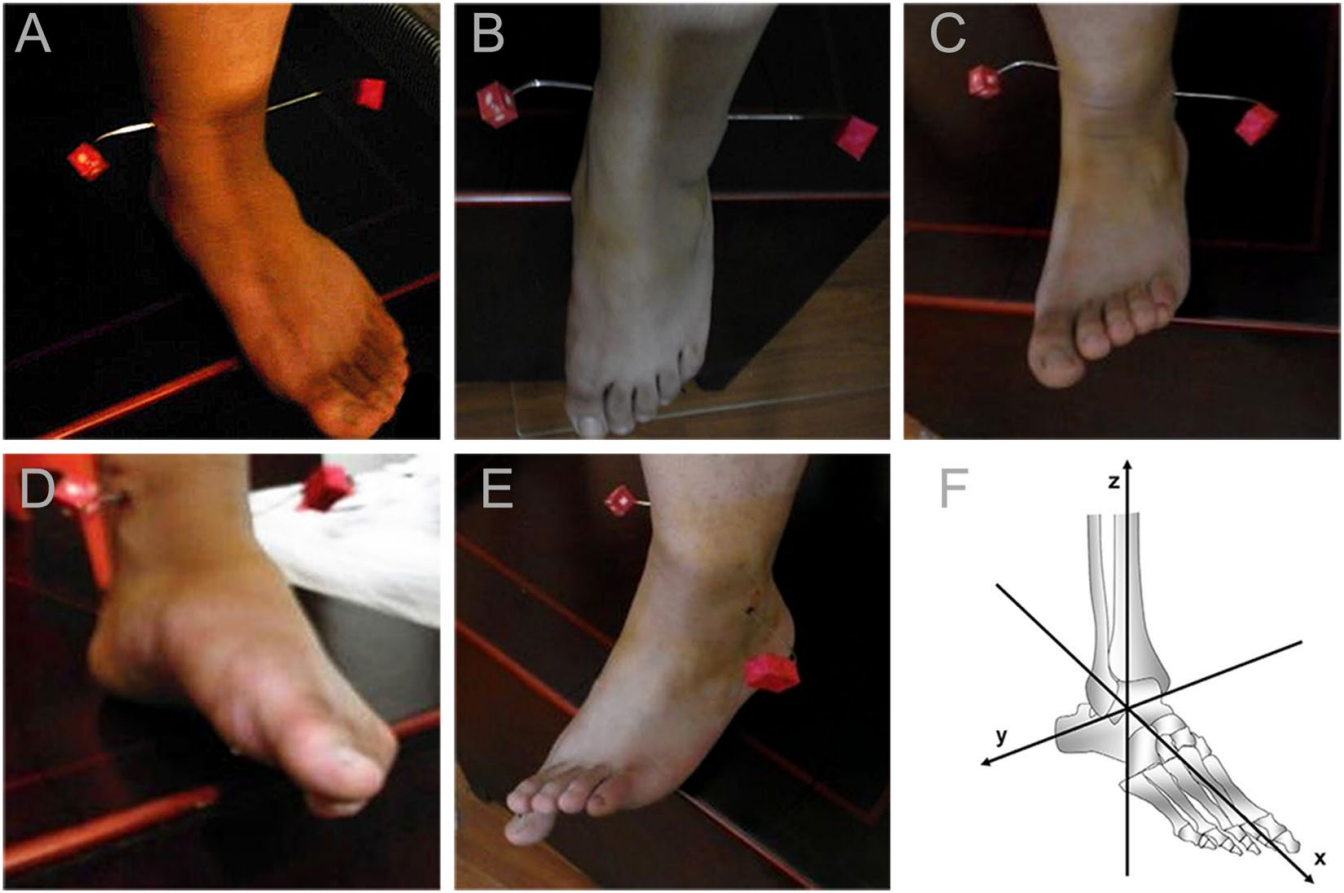
Ankle joint underwent 3D laser scanning *in vivo*. (**A**) the anatomical position, (**B**) ankle plantar flexion, (**C**) ankle dorsiflexion, (**D**) ankle inversion, (**E**) ankle eversion, (**F**) the x-y-z coordinate system used to describe the bone positon.

We invited a randomly selected subcohort of 6 participants in each shape of the DTS to undergo 3D laser scanning (3D Digital Corporation, USA), to provide the micro-movement characteristic of the DTS. The participant sat on a chair, and the cortical bones of the medial and lateral malleolar were fixed with two imaging detection units at the junction of the transverse (x-y plane, 9 mm proximal to the tibial plafond) and frontal (y-z plane). Then the 3D laser scanning tested the displacement of two imaging detection units in the different positions of ankle joint: the anatomical position; plantar flexion 10°, 20°, 30° and 45°; dorsiflexion 10° and 20°; inversion 10°, 20° and 30°; and eversion 10° and 20°. Computer based on the positions of the two imaging detection units were used to calculate the displacement in the x, y and z axis (Fig. 1).

### Clinical assessment of the incidence rates of recurrent lateral ankle sprain

The assessment procedure was incident recurrent lateral ankle sprain of different shapes of the DTS during the three years period of study in the 300 young athletic trainers. In the trial monitoring, all participants were asked to attend our clinic to check immediately after ankle injury.

### Statistical Analysis

Before the statistical analyses, all outcome variables were recorded into dichotomies. Age, weight, height, and body mass index (BMI) were recorded as above or below the median value. SPSS 19.0 (SPSS Company, America) statistical software package was taken for statistical analysis. *P* < 0.05 indicates significant difference, while *P* < 0.01 refers to extremely significant difference.

## Results

**Figure 2:**
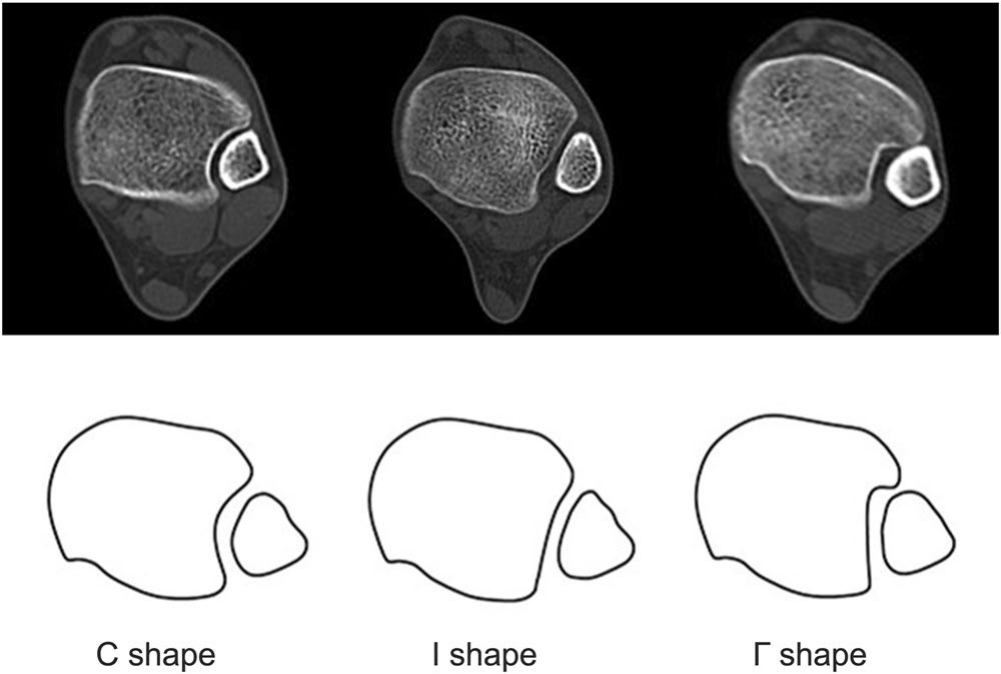
Illustrations of the DTS classification system in 3 different types based on the morphology of incisura fibularis.

In the 300 young physical training soldiers, there were 231 male and 69 female, aging from 18 to 20 years with a mean age of 18.6 years. The shapes of the DTS were organized into 3 distinct types based on the morphology of incisura fibularis. Type 1 was defined as “C” shape with giving the syndesmosis a crescent shape. Type 1 accounted for 168 of 300 participants included in this study (56%). There were 128 male and 40 female. Type 2 was defined as “1” shape with the incisura fibularis was shallow. There were 76 participants (25%), with 59 male and 17 female. Type 3 was defined as “Г” shape with the incisura fibularis was shallow while the anterior or posterior tibial tubercle had a larger protrusion, and accounted for 56 of 300 participants (19%). There were 44 male and 12 female. No significant differences in baseline characteristics were found between the three groups (Table 1 and Fig. 2).

**Table 1:** Demographic Information for Each Type Included in the Shapes of the DTS.

Parameter	C Shape	1 Shape	Г Shape
Overall, n (%)	168 (56)	76 (25)	56 (19)
Male/ female, n	128/40	59/17	44/12
Mean age (range), yr	18.5 ± 0.4 (18–20)	18.7 ± 0.5 (18–20)	18.8 ± 0.5 (18–20)
Right/ left ankle involved, n	97/71	43/33	31/25
BMI, kg/cm^2^	24.8 ± 3.1 (21.3–27.2)	24.5 ± 2.6 (21.7–26.5)	24.2 ± 2.8 (21.5–26.7)
Number of sprains	4.3 ± 0.4 (3–6)	5.2 ± 0.4 (3–7)	3.7 ± 0.3 (3–4)
Exam findings			
ATFL injury	4	11	2
bone avulsion	0	4	0
Hindfoot alignment			
Normal alignment	7	13	3
Abnormal alignment	0	0	0

BMI, body mass index.

The analysis results of the 18 participants in the subcohort (6 cases in each shape) are shown in Table 2. When the ROM of ankle plantar flexion was regularly increased, the displacement of each shape type in the x and y axis was gradually increased, but the displacement in the z axis showed no significant change. The “1” shape showed the widest range of displacement but was not statistically different from the “C” and “Г” shapes (0.05 < *P* < 0.1). When the ROM of ankle dorsiflexion was regularly increased, the displacement of each shape type in the x and y axis was gradually increased, but the displacement in the z axis showed no significant change. The “1” shape exhibited significantly increased displacement in the y axis, during ankle dorsiflexion was 20°, compared with the “C” and “Г” shapes (*P* < 0.01). When the ROM of ankle inversion was regularly increased, the displacement of each shape type in the x, y and z axis was gradually increased. Comparing with the “C” and “Г” shapes, the “1” shape had a significantly increased displacement in the y axis, along with the ROM of ankle inversion on the position more than 20° (*P* < 0.01). When the ROM of the ankle eversion was regularly increased, the displacement of each shape type in the y and z axis was gradually increased, but the displacement in the x axis was gradually decreased. Comparing with the “C” and “Г” shapes, the “1” shape had a significantly increased displacement in the y axis, when the ankle eversion was 20° (*P* < 0.01).

**Table 2:** Results of Displacement of the DTS on Different Positions in Each Shape Type (mm).

Position	C Shape			1 Shape			Г Shape		
	x-axis	y-axis	z-axis	x-axis	y-axis	z-axis	x-axis	y-axis	z-axis
Ankle plantar flexion									
10°	0.19 ± 0.04	0.22 ± 0.06	−0.62 ± 0.12	0.23 ± 0.05	0.27 ± 0.05	−0.71 ± 0.11	0.18 ± 0.03	0.21 ± 0.04	−0.65 ± 0.13
20°	0.46 ± 0.05	0.63 ± 0.12	−0.64 ± 0.09	0.52 ± 0.05	0.69 ± 0.11	−0.72 ± 0.17	0.44 ± 0.04	0.65 ± 0.09	−0.65 ± 0.17
30°	0.61 ± 0.07	0.84 ± 0.19	−0.66 ± 0.11	0.67 ± 0.06	0.90 ± 0.21	−0.74 ± 0.14	0.62 ± 0.04	0.82 ± 0.17	−0.66 ± 0.12
45°	0.92 ± 0.11	1.07 ± 0.13	−0.67 ± 0.16	0.98 ± 0.09	1.18 ± 0.15	−0.74 ± 0.19	0.94 ± 0.07	1.03 ± 0.11	−0.68 ± 0.16
Ankle dorsiflexion									
10°	−0.57 ± 0.04	0.58 ± 0.11	0.23 ± 0.03	−0.61 ± 0.07	0.67 ± 0.12	0.27 ± 0.04	−0.59 ± 0.05	0.62 ± 0.14	0.21 ± 0.04
20°	−0.76 ± 0.07	0.92 ± 0.17	0.26 ± 0.06	−0.82 ± 0.07	1.23 ± 0.16^*^	0.33 ± 0.07	−0.77 ± 0.06	0.93 ± 0.18	0.24 ± 0.05
Ankle inversion									
10°	0.74 ± 0.05	0.85 ± 0.07	−0.29 ± 0.03	0.79 ± 0.07	1.01 ± 0.11	−0.34 ± 0.05	0.73 ± 0.04	0.87 ± 0.05	−0.30 ± 0.03
20°	0.82 ± 0.05	1.23 ± 0.12	−0.47 ± 0.06	0.88 ± 0.06	1.52 ± 0.18^*^	−0.55 ± 0.04	0.83 ± 0.06	1.29 ± 0.14	−0.45 ± 0.05
30°	0.96 ± 0.13	1.62 ± 0.15	−0.59 ± 0.04	1.13 ± 0.15	2.11 ± 0.19^*^	−0.67 ± 0.07	1.02 ± 0.09	1.68 ± 0.16	−0.56 ± 0.07
Ankle eversion									
10°	−0.76 ± 0.12	0.77 ± 0.10	−0.18 ± 0.03	−0.85 ± 0.14	0.84 ± 0.12	−0.21 ± 0.04	−0.73 ± 0.09	0.75 ± 0.11	−0.16 ± 0.03
20°	−0.44 ± 0.07	0.96 ± 0.14	−0.47 ± 0.06	−0.48 ± 0.11	1.19 ± 0.17^*^	−0.52 ± 0.07	−0.45 ± 0.08	0.94 ± 0.15	−0.41 ± 0.05

*P < 0.01.

**Figure 3:**
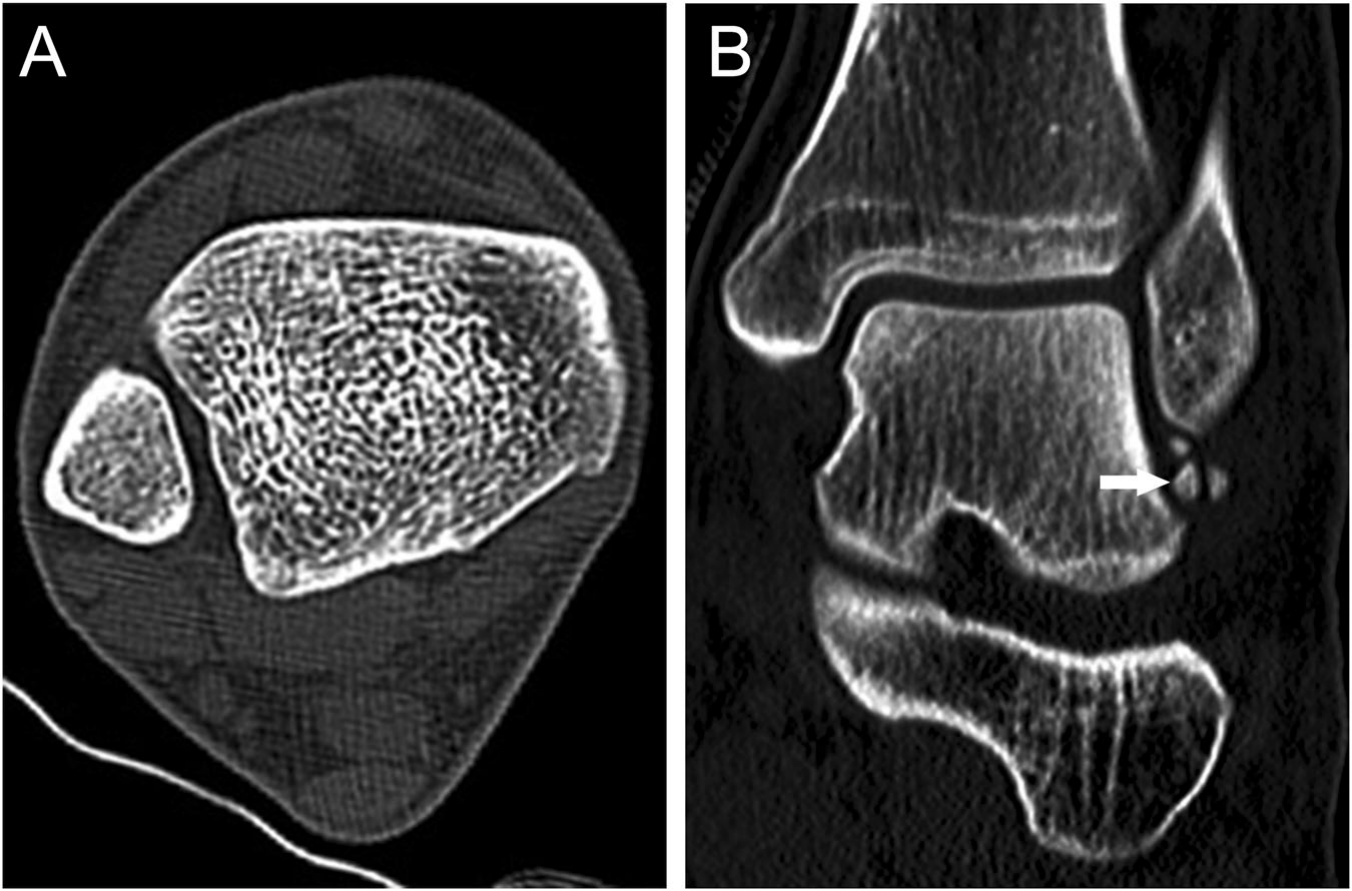
A 19-year-old man with recurrent lateral ankle sprain and ATFL injury with avulsed bone fragments. (**A**) Axial CT image showed that the shape of the DTS was the “1” shape. (**B**) Coronal CT image demonstrated bone avulsion (white arrow) from the ATFL injury. The bone fragments were likely to represent an old fracture.

During the three years period of study, 39 participants suffered ankle sprain, and 23 cases experienced recurrent lateral ankle sprains. Of the 23 recurrent lateral ankle sprains, 7 participants of the incisura fibularis were the “C” shape, 13 participants were the “1” shape, and 3 participants were the “Г” shape. The “1” shape of the incisura fibularis showed highest risk in the three shapes in incident recurrent lateral ankle sprains (*P* < 0.05). There were 4 participants with ATFL injury with avulsed bone fragments in the “1” shape (Fig. 3). However, it did not occur in the “C” and “Г” shapes (Table 1).

## Discussion

This study demonstrated that it was possible to classify the shapes of the DTS based on the morphology of incisura fibularis. We were able to classify shapes of the DTS in three shape types: “C” shape (type 1) with giving the syndesmosis a crescent shape, “1” shape (type 2) with the incisura fibularis was shallow, and “Г” shape (type 3) with the incisura fibularis was shallow while the anterior or posterior tibial tubercle had a larger protrusion. The most common shape of the DTS is the “C” shape, followed by the “1” and “Г” shape. The “1” shape of the DTS causes a widening of the ankle mortise with ankle motion, and people with this shape may have more risk of recurrent lateral ankle sprains and ATFL injury with avulsed bone fragments.

The stability of the ankle mortise is enhanced because the dome-shaped body of the talus fits snugly into the slightly concave tibial undersurface. However, the body of the talus is wedge-shaped from front to back being wider anteriorly, and thinner posteriorly. When the foot is moved from a dorsiflexed position to a plantar-flexed position, this motion causes a widening of the ankle mortise and results in the risk of ankle sprain. Ankle ligaments provide mechanical stability, proprioceptive information, and directed motion for the joint. Much research has focused on injury to the lateral ankle ligaments and inversion ankle sprains^32–34^. Risk factors are those activities, such as basketball and jumping sports, in which an athlete can come down on and turn the ankle or step on an opponent’s foot. Increased BMI and heel height are also the more risk factors for ankle sprain^35–38^. In the meantime, the anatomical predisposition of the ankle joint is another risk factor for the lateral ankle sprain^39^. In those who have had a sprain in the past, it is also easier to turn the ankle and cause a new sprain. Therefore, one of the major risk factors of spraining the ankle is having instability. The DTS is one of the important structural components to maintain the integrity of the ankle mortise and act to statically stabilize the ankle joint. However, literature on the associations between shapes of the DTS and risk of ankle sprain has not yet been reported.

Much is known about the patterns of the DTS activation that physiological movement in the 3D space^19, 21, 23, 27^. However, previous studies of the biomechanical properties of the DTS are limited in the vitro anatomy and the resting state, and the actual biomechanical properties of the DTS are not completely understood. The DTS and ankle joint are biomechanically linked. It is not possible for the tibiotalar, DTS, or subtalar joints alone to produce the entire arc of ankle motion. In this study, we calculated the displacement of the DTS in 3D space underwent 3D laser scanning *in vivo*. Of this examination, the imaging detection units were fixed at the junction of the transverse and the frontal in the cortical bones of medial and lateral malleolar. This method could exclude the muscles and ligaments from an interference movement, and could be a true reflection of the physiological movement of the DTS in 3D space.

According to our study results, the activation and ROM of the DTS are not only influenced by the ROM of ankle joint, but also by the depth of the incisura fibularis and the shape of the tibial tubercles. The width of the ankle mortise will vary with the different depth of the incisura fibularis and shape of the tibial tubercles during the changes of gait cycle. When the ankle mortise space is widening, the ankle mortise turns more unstable, resulting in the risk of ankle sprain^5, 6^. In our study, the “1” shape showed widest range of displacement of the DTS in the y-axis, along with the ROM on the position more than 20° of the ankle dorsiflexion, inversion and eversion. These cause a widening of the mortise, resulting in the risk of ankle instability^5, 6^. As a result of 3 years follow-up in the 300 participants, the “1” shape of the DTS had a higher risk of recurrent lateral ankle sprains and ATFL injury with avulsed bone fragments, and this result consistent with the result of the displacement of the DTS in 3D space underwent 3D laser scanning. As noted above, we considered that the rotation axis of “C” shape lies along the center of the incisura fibularis, and “1” shape lies along the anterior or posterior tibial tubercle. It is possible that the “1” shape caused the increased width of the ankle mortise and the strain of the ATFL as the ankle motion.

We acknowledge some limitations to this study. First, it was a single-site study at an academic medical center. Future prospective randomized studies with appropriate sample size need to detect the morphology and micro-movement characteristic of the DTS. Furthermore, these participants also need to gait analysis for the different shapes of the DTS. Another weakness is that no other possible risk factors were considered, such as injury mechanism, activity level and ligamentous laxity, etc.

## Conclusion

In summary, it was possible to classify the shapes of the DTS according to the morphology of incisura fibularis. We were able to classify shapes of the DTS in three shape types: “C” shape, “1” shape and “Г” shape. The most common shape of the DTS is the “C” shape, followed by the “1” and “Г” shape. People with “1” shape of the DTS may have more risk of recurrent lateral ankle sprains.

### Ethics Approval

Xiamen University Ethical Review Committee.
